# Direct Segmentation of Mammography and Tomosynthesis Sinograms for Lesion Localization

**DOI:** 10.3390/tomography12030034

**Published:** 2026-03-03

**Authors:** Estefanía Ruíz Muñoz, Leopoldo Altamirano Robles, Raquel Díaz Hernández, Kelsey Alejandra Ramírez Gutiérrez, Saúl Zapotecas-Martínez, José de Jesús Velázquez Arreola

**Affiliations:** 1 Instituto Nacional de Astrofísica, Óptica y Electrónica, Sta. María Tonantzintla, Puebla 72840, Mexico; estefania.ruiz@inaoe.edu.mx (E.R.M.); raqueld@inaoep.mx (R.D.H.); kramirez@inaoe.mx (K.A.R.G.); szapotecas@inaoep.mx (S.Z.-M.); josej.velazqueza@inaoep.mx (J.d.J.V.A.); 2 Secretaria de Ciencia, Humanidades, Tecnología e Innovación (SECIHTI), Mexico City 03940, Mexico

**Keywords:** sinograms, lesion segmentation, mammography, digital breast tomosynthesis, U-Net

## Abstract

**Background**: The Detection and localization of breast lesions remain challenging in mammography and digital breast tomosynthesis (DBT) due to tissue overlap and information loss during volumetric reconstruction. Sinograms preserve the full angular projection data acquired during scanning, enabling analysis of tissue structure without reconstruction. **Methods**: This study proposes a direct segmentation approach for mammography and DBT sinograms using a U-Net architecture. Experiments were conducted on 1082 annotated mammography mass images from the CBIS-DDSM dataset (521 benign, 561 malignant) and 272 annotated DBT images from the Breast Cancer Screening DBT dataset (136 benign, 136 malignant). Dataset splitting was performed at the patient level to prevent data leakage, and all reported quantitative results correspond to the independent test set, with the validation set used solely for model selection and early stopping. Three input configurations were evaluated: mammography sinograms, DBT sinograms, and a combined model. **Results**: The mammography model achieved the highest performance (Dice: 0.94 training, 0.90 test), outperforming DBT alone (0.77 training, 0.70 test). Multimodal fusion improved DBT results (Dice: 0.84 test). Centroid analysis showed 99.11% correspondence with reference annotations (average distance: 2.83 pixels), and partial back-projection reconstructions confirmed anatomical consistency. Compared with YOLOv5x, the proposed approach provided superior lesion localization, particularly for small or multiple lesions. **Conclusions**: Direct sinogram segmentation is an efficient, clinically viable strategy for breast lesion detection and localization.

## Simple Summary

Breast cancer detection commonly relies on mammography and digital breast tomosynthesis, but lesion localization is often hindered by tissue overlap and information loss during image reconstruction. This study investigates a direct analysis of sinograms, which preserve raw projection data, without prior reconstruction. A deep learning-based segmentation approach is proposed to localize breast lesions directly from sinograms. Mammography sinograms achieved the most accurate localization, while combining mammography and tomosynthesis sinograms improved tomosynthesis performance, supporting more reliable lesion localization.

## 1. Introduction

Breast cancer is one of the leading causes of mortality worldwide [1]. Early detection and timely diagnosis are key factors in reducing the mortality rate associated with this disease. In this context, Digital Breast Tomosynthesis (DBT) has emerged as an advanced medical imaging technique that overcomes several limitations of conventional mammography, particularly tissue superposition, which can obscure the presence of lesions [2].

DBT acquires multiple projections of the breast from different angles along a limited arc, generating a set of images that are subsequently used to reconstruct sectional slices of the breast tissue [3]. These projections can be arranged into a representation known as a sinogram, which encodes spatial and angular information about the internal structures of the breast. Although a sinogram is typically considered an intermediate representation, it preserves key features of lesions and surrounding anatomy [4], making it a potentially valuable source of information for computer-aided diagnosis (CAD) [5].

Most segmentation and classification studies in DBT have traditionally focused on reconstructed volumes. However, the reconstruction process is computationally demanding and may introduce artifacts that compromise image quality and diagnostic reliability [6]. Motivated by these limitations, recent research has explored the feasibility of working directly on sinograms, bypassing the reconstruction stage. This strategy can reduce computational cost, simplify clinical workflows, and better preserve the raw information captured by the scanner [7].

Although sinograms preserve the original angular information, intrinsic differences between mammography and DBT acquisitions may affect segmentation performance. Mammography is acquired using a full-angle geometry with a single high-quality projection per view, producing more stable sinograms with lower noise variability. In contrast, DBT relies on limited-angle acquisition and multiple low-dose projections, which generate noisier and partially incomplete sinograms, particularly along the angular dimension, potentially making direct segmentation more challenging. Furthermore, in this study the number of annotated DBT images is considerably smaller than that of mammography, which also influences the generalization capability of the models and must be considered when interpreting comparative results. The novelty of this work does not lie in claiming clinical superiority between modalities, but in demonstrating that lesion segmentation and localization can be achieved directly in the sinogram domain without volumetric reconstruction, and that even limited-angle DBT sinograms retain sufficient information for accurate localization.

Building on this perspective, the present study investigates direct lesion segmentation from sinograms using a U-Net architecture. The proposed approach evaluates different input configurations and assesses their potential to simplify and accelerate breast image analysis without requiring full volumetric reconstruction. The main contributions of this study are summarized as follows:

- Evaluation of a U-Net model for direct lesion segmentation from sinograms, comparing its performance on mammography sinograms, DBT sinograms, and a combined representation.
- Exploration of partial reconstructions obtained from segmented sinograms to estimate lesion locations, even when their morphology is not fully recovered.
- Analysis of the strengths and limitations of each input configuration, highlighting the potential of sinograms for developing faster and more efficient image analysis systems without prior reconstruction.

The remainder of this article is structured as follows: Section 2 reviews related work on segmentation in mammography, DBT, and sinogram-based imaging. Section 3 describes the representation and processing of sinogram images in the context of breast imaging. Section 4 details the methodology, including preprocessing steps, the U-Net architecture employed, and the evaluated input configurations. Section 5 presents the experimental results, including quantitative and qualitative evaluations of segmentation and localization performance. Section 6 discusses the key findings, clinical relevance, and limitations of the study. Finally, Section 7 provides concluding remarks and outlines directions for future research.

## 2. Related Work

### 2.1. Segmentation in Reconstructed Images

Breast lesion segmentation using deep learning has attracted significant attention in recent years, particularly through convolutional architectures such as U-Net. These models are widely used in medical image segmentation due to their capacity to capture both high- and low-resolution spatial information.

In the context of DBT, an emerging line of research focuses on detecting masses directly from three-dimensional volumes. Zhou et al. [8] proposed a 3D Mask R-CNN for breast lesion segmentation in DBT, achieving a sensitivity of 90% and a false-positive average of 0.8, outperforming 2D image-based methods.

In other imaging modalities, such as ultrasound, advanced U-Net variants integrating attention mechanisms and deeper architectures have been developed. In [9], AAU-Net was introduced as a hybrid network combining spatial and channel attention, improving segmentation performance in images with complex tumor morphology. Similarly, RCA-IUNet [10] incorporated cross-attention and Inception-like modules to enhance segmentation robustness across tumors of varying scales in clinical settings.

In mammography, several studies have adapted U-Net-based architectures to optimize segmentation performance on 2D images. Haq et al. [11] introduced U-Net 3+, which achieved a Dice coefficient of 98.47% on the INbreast dataset. UGGNet, a hybrid model combining U-Net and VGG16, achieved 78.2% classification accuracy on ultrasound images. More recently, UltraSegNet [12] combined attention mechanisms with multiscale feature fusion, achieving a Dice score of 91.1% and an accuracy of 91.5% on the BUSI dataset.

### 2.2. Segmentation in the Sinogram Domain

Despite the advances in reconstructed images, most current approaches rely on mammograms or DBT volumes. Sinograms, which represent the raw projections acquired before reconstruction, have received limited attention despite their potential benefits, including reduced vulnerability to reconstruction artifacts, lower computational cost, and preservation of original acquisition information.

Ruiz Muñoz et al. [13] evaluated DBT-derived sinograms for breast lesion classification using ResNet18 and ResNet50, achieving accuracy, sensitivity, and F1-scores up to 94.96%. Similarly, the use of U-Net on sinograms has been explored in micro-computed tomography, demonstrating notable improvements in reconstruction quality and significant artifact reduction [14].

These studies suggest that analyzing sinograms directly using deep learning is both feasible and promising. However, this line of research remains at an early stage and requires further validation. The present work contributes to this direction by systematically evaluating lesion segmentation from mammography sinograms, DBT sinograms, and a combined input configuration, with an emphasis on lesion localization.

Table 1 summarizes recent deep learning approaches for breast lesion segmentation and classification, highlighting their architectures, imaging modalities, and performance metrics. This comparison underscores the scarcity of direct sinogram-based methods and motivates the approach proposed in this study.

## 3. Sinograms Representation and Processing in Digital Breast Imaging

This section explains the theoretical and methodological bases for generating and analyzing sinograms in digital breast imaging. The principles of sinogram construction, their relation with acquisition processes in mammography and DBT, and the strategies applied for their processing are described. The purpose is to establish the conceptual basis supporting the use of sinograms as an intermediate representation for lesion detection and localization, without the need for complete volumetric reconstruction.

### 3.1. Digital Breast Tomosynthesis (DBT)

Digital Breast Tomosynthesis (DBT) is an advanced medical imaging modality designed to improve the detection of breast lesions by reducing tissue superposition, a major limitation of conventional mammography.

In DBT, multiple low-dose projections of the breast are acquired over a limited angular range (typically between $\pm 15^{\circ }$ and $\pm 50^{\circ }$) using a flat-panel digital detector. These two-dimensional projections are subsequently processed through a reconstruction algorithm to generate quasi–quasi-three-dimensional sectional images of the breast tissue, enabling improved visualization of internal anatomical structures [15].

### 3.2. Sinogram Generation Using the Radon Transform

A sinogram is a graphical representation that organizes the projections acquired from multiple angles during a DBT scan prior to volumetric reconstruction. In this representation, each row corresponds to a specific projection angle, and each column captures the detector intensity at that angle [16].

Mathematically, a sinogram is constructed using the Radon Transform. For a two-dimensional function f(x,y) representing the tissue density distribution, the Radon Transform Rf(θ,t) is defined as:

(1) $$Rf\left(\theta ,t\right)=\int_{-\infty }^{\infty }f\left(t\cos\theta -s\sin\theta ,\;t\sin\theta +s\cos\theta \right)\;ds$$

Here, Rf(θ,t) denotes the sinogram value at projection angle θ and detector position *t* [17]. This formulation encodes the spatial structure of the breast into the angular projection domain, serving as the foundation of classical reconstruction algorithms.

Although DBT uses a limited number of acquisition angles—unlike full-scan computed tomography—sinograms preserve essential information about internal structures such as masses and microcalcifications. Consequently, they can be used for diagnostic and computational analysis tasks without requiring full volumetric reconstruction [18].

### 3.3. Image Reconstruction from Sinograms

Image reconstruction from a sinogram aims to recover the original function f(x,y) from its projections Rf(θ,t). The most widely used reconstruction technique is the Filtered Back-Projection (FBP) algorithm, which applies a frequency-domain filter to each projection and subsequently backprojects the filtered data into the spatial domain [17,19]. The general formulation of FBP is expressed as:

(2) $$f\left(x,y\right)=\int_{0}^{\pi }{\overset{ˇ}{R}}_{f}\left(\theta ,\;x\cos\theta +y\sin\theta \right)\;d\theta$$

where ${\overset{ˇ}{R}}_{f}\left(\theta ,t\right)$ denotes the filtered projection at angle θ and position *t*.

In DBT, however, the limited number of acquisition angles and the constrained system geometry introduce several challenges. These constraints commonly result in reconstruction artifacts and reduced spatial resolution, which can hinder the clear visualization of subtle lesions. Although traditional reconstruction techniques can generate sectional slices, they often introduce distortions when the projection set is sparse and may also be computationally demanding.

These limitations have motivated the exploration of alternative strategies that rely on directly analyzing the information contained in sinograms. Such approaches aim to improve diagnostic efficiency while avoiding the drawbacks associated with volumetric reconstruction.

### 3.4. Lesion Analysis in the Sinogram Domain

In a sinogram, a vertical stripe represents the variation of a specific line in the spatial domain as the acquisition angle changes. Conversely, a horizontal stripe corresponds to the intensity recorded by a particular detector element across all projections. Under this relationship, a localized lesion in the spatial domain appears as a continuous curve within the sinogram, whose trajectory is determined by its geometric location and structural properties [17].

This correspondence enables the inference of lesion location directly from the sinogram, without requiring full volumetric reconstruction. Such characteristics have encouraged the development of deep learning approaches that take sinograms as direct input for tasks such as classification and segmentation.

**Figure 1:**
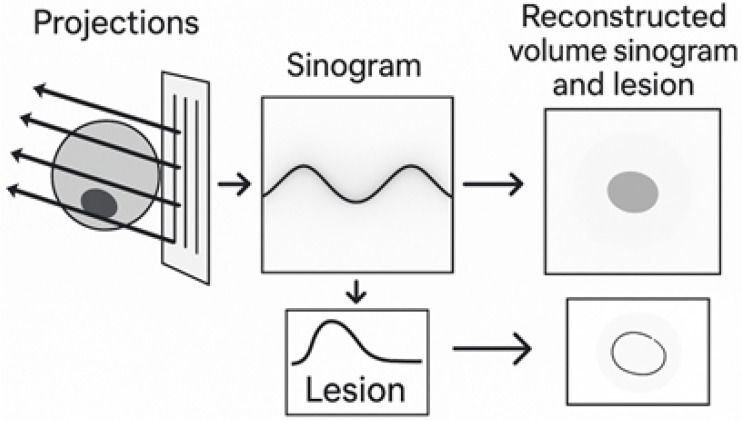
Process of sinogram generation in digital breast imaging. Author’s own work.

Figure 1 presents a schematic overview of the sinogram acquisition and analysis process. Multiple angular projections are first acquired to capture variations in tissue attenuation. These projections are then assembled into a sinogram, where the horizontal axis represents the acquisition angle and the vertical axis corresponds to the recorded intensity along each detector line.

In this representation, lesions appear as distinctive curves that reflect their angular behavior. Based on this information, reconstruction techniques such as Filtered Backprojection (FBP) may be applied to obtain sectional images or volumes when required. This figure summarizes the concepts discussed in Section 3 and highlights how sinograms enable lesion characterization and localization before reconstruction, making this approach suitable for both DBT and projection mammography.

### 3.5. Relevance of Direct Segmentation on Sinograms

The direct use of sinograms in deep-learning-based segmentation models offers several advantages. First, it eliminates the reconstruction step, which considerably reduces computational time. Second, avoiding backprojection helps minimize artifacts that typically arise during DBT image reconstruction. Third, this approach enables the analysis of raw acquisition data, improving traceability and preserving the integrity of the original information.

Despite these advantages, sinogram-based analysis requires the development of specialized algorithms capable of interpreting its angular structure and the domain’s intrinsic characteristics. These challenges motivate the exploration of tailored deep learning architectures for accurate lesion segmentation directly in sinogram space.

## 4. Methodology

**Figure 2:**
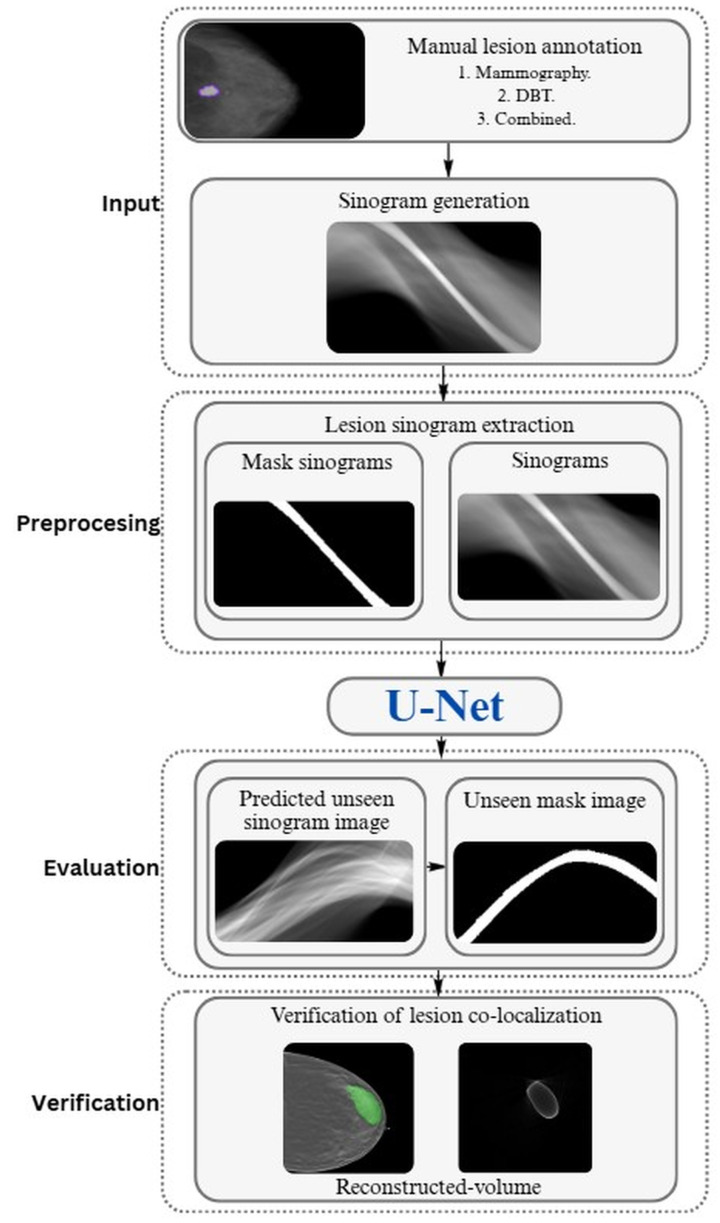
Workflow for direct lesion segmentation from sinograms. Author’s own work.

This section provides a detailed description of the implementation and evaluation procedures applied in the proposed approach (Figure 2). It includes the stages of sinogram generation and preprocessing; dataset configuration; segmentation architecture; and training, evaluation, and reconstruction strategies. The objective is to establish a clear and reproducible workflow for assessing the performance of direct segmentation methods applied to mammography and DBT sinograms.

### 4.1. Input and Sinogram Generation

This investigation presents a method based on a U-Net architecture to segment breast lesions directly from sinograms generated from mammograms and DBT slices. To evaluate the effectiveness of the model, three different input configurations were considered:

1. Sinograms obtained from conventional mammograms.
2. Sinograms generated from DBT slices.
3. Combined set of sinograms from mammography and DBT.

The process begins with the manual annotation of lesions in the original clinical images. Based on these annotations, the corresponding sinogram is generated, and lesion-centered regions are extracted using the annotation masks. These regions are then processed to train a U-Net architecture, which performs segmentation directly in the sinogram domain. Finally, the reconstructed segmented masks can be obtained through backprojection, enabling the evaluation of their spatial localization within the original volume.

Clinical images from the CBIS-DDSM [20] and Breast Cancer Screening DBT [21] datasets were used, which include expert annotations of benign and malignant lesions. For clarity, the datasets were configured as follows: from the CBIS-DDSM mammography dataset, which contains 2620 studies from 1566 participants, we used the subset with lesion annotations corresponding to 1082 mass images (521 benign and 561 malignant). From the Breast Cancer Screening DBT dataset, although the repository includes images from 5060 subjects, only 272 images provided publicly available lesion annotations suitable for segmentation; therefore, our experiments were conducted on this annotated subset (136 benign and 136 malignant).

To ensure rigorous evaluation and prevent data leakage, dataset division was performed at the patient level, guaranteeing that images from the same patient did not appear in multiple subsets. Moreover, both imaging modalities from the same patient were consistently assigned to a single subset.

The images and masks were loaded as pairs using a custom PyTorch (version 2.6.0) Dataset, resized to 128×128 pixels, and normalized to the range [0, 1]. To improve generalization, data augmentation techniques were applied during training, including random rotations (±$15^{\circ }$), horizontal flipping, and Gaussian noise. DataLoaders were created with a batch size of 16 for both training and validation sets.

All quantitative metrics were reported on the independent test set, while the validation set was used exclusively for model selection and early stopping. This setup ensures a reproducible and statistically robust evaluation of lesion segmentation in both mammography and DBT sinograms.

#### Sinogram Generation

Sinograms were generated by applying the Radon Transform to mammograms and individual DBT slices. Angular projections were computed within a range of $\pm 25^{\circ }$, using increments of $2.5^{\circ }$. In the resulting sinogram, the horizontal axis corresponds to the projection angles, and the vertical axis represents the accumulated intensity at each angle.

Since raw projections are not accessible from commercial mammography or DBT systems, the sinograms were generated from the available reconstructed images. Although these sinograms do not correspond to the original acquisition domain, this procedure preserves the linearity of the projections and the structural characteristics of the lesions. To ensure consistency and validity, the segmentation results obtained from the sinograms were verified against the original reconstructed images.

All images were normalized to the range [0, 1] and resized to 256×256 pixels to standardize the model inputs. This preprocessing step ensures that the input data is compatible with the deep learning models while preserving relevant lesion information in both the spatial and projection domains.

### 4.2. Preprocessing and Lesion-Focused Sinogram Extraction

The preprocessing phase begins by resizing the original reconstructed images to a uniform resolution of 256 × 256 pixels. Next, the complete sinograms are generated from these resized images using the Radon transform. Afterward, lesion-centered regions are extracted from the sinograms using the annotation masks, generating input pairs that consist of the image sinogram and its corresponding mask sinogram.

Both images and masks are normalized to an intensity range between 0 and 1. To enhance the model’s generalization capability, data augmentation techniques are applied during training, such as random rotations of up to ±$15^{\circ }$ and horizontal flipping.

This strategy allows the network to learn patterns directly from the sinograms, preserving the geometric information associated with the projections while focusing on lesion-specific regions. Additionally, it facilitates comparisons across different input configurations.

#### Input Configurations

Three experimental configurations were evaluated, designed to analyze the impact of the source image type on the model’s performance:

1. Mammography data: Training and testing conducted exclusively with sinograms generated from mammography images.
2. DBT data: Training and testing conducted exclusively with sinograms obtained from DBT images.
3. Combined data: A single training set combining sinograms from both mammography and DBT.

In all cases, each sinogram was processed as an independent single-channel image, without using multichannel inputs. This choice enabled evaluating how the type of source image affects model performance and segmentation quality.

### 4.3. Model Architecture and Segmentation Training

The segmentation task was performed in a standard U-Net architecture, chosen for its ability to capture both local features and more general contextual information within the sinogram domain. The network follows a symmetric encoder–decoder structure with skip connections that facilitate the recovery of fine spatial details.

- Encoder: The encoder consists of four convolutional blocks, each composed of two-dimensional convolutions with a 3×3 kernel, followed by Batch Normalization, ReLU activation, dropout (p=0.2), and 2×2 MaxPooling to reduce the spatial resolution progressively.
- Decoder: The decoder replicates the encoder structure and includes four upsampling blocks, which consist of an upsampling step followed by a convolution, complemented by skip connections that transfer high-resolution features and assist in reconstructing spatial detail.
- Output Layer: Finally, 1×1 convolutional layer with sigmoid activation is used to generate the binary probability mask.

The model was implemented in PyTorch using an input size of 128×128 pixels. The network was trained from scratch for each of the evaluated configurations. No pre-trained weights were employed.

#### 4.3.1. Model Training

Each configuration was trained for 100 epochs using a consistent optimization and validation scheme. The following parameters defined the training process:

- Optimizer: Adam with a learning rate of $1\times 10^{-4}$ and L2 regularization (weight_decay = $1\times 10^{-6}$).
- Loss function: A hybrid objective combining Dice loss and Binary Cross-Entropy (BCE), designed to balance pixel-wise accuracy and overlap consistency.
- Batch size: 16, selected to provide a suitable trade-off between computational efficiency and gradient stability.
- Model selection: For each experiment, the model achieving the highest validation Dice score was retained.

Model performance was evaluated using standard segmentation metrics, i.e., the Dice Score and Intersection over Union (IoU), complemented by qualitative inspection of the predicted masks. This dual evaluation enabled us to assess both numerical performance and the visual consistency of segmentations across anatomical variations.

Additional training details: To provide further reproducibility, the following information is added:

- Early stopping: The model was trained for a maximum of 100 epochs without formal early stopping. The final model was selected based on the highest validation Dice, which for the DBT dataset was achieved at epoch 100 (Dice = 0.7011), indicating stable convergence.
- Learning rate scheduling: A fixed learning rate was used throughout training, as preliminary experiments showed stable convergence without scheduling.
- Hardware and training time: Training was performed on an NVIDIA GeForce RTX 3060 GPU with 12 GB of VRAM. Each training configuration took less than 30 min due to the relatively small image size (128 × 128 pixels) and batch size (16).

##### Dice Score

The Dice Score quantifies the degree of overlap between the predicted mask *P* and the ground truth mask *G* [22]:

(3) $$\mathrm{Dice}=\frac{2\cdot |P\cap G|+\epsilon }{|P|+|G|+\epsilon }$$

where:

- *P*: Predicted binary mask.
- *G*: Ground truth mask.
- |P∩G|: Number of pixels in the intersection of *P* and *G*.
- |P| and |G|: Number of positive pixels in *P* and *G*, respectively.
- ϵ: Small constant ($10^{-5}$) to avoid division by zero.

Values close to 1 indicate a high agreement between prediction and ground truth, while values near 0 indicate poor overlap.

##### Intersection over Union (IoU)

IoU measures the ratio between the intersection and the union of the predicted and reference masks [22]:

(4) $$\mathrm{IoU}=\frac{\left|P\cap G\right|}{\left|P\cup G\right|}$$

where:

- |P∩G|: Pixels shared by both masks.
- |P∪G|: Total pixels present in at least one of the masks.

IoU is a more traditional metric than Dice, penalizing both false positives and false negatives.

##### BCE + Dice Loss

Model optimization was performed using a combined loss function that integrates Binary Cross-Entropy (BCE) and Dice Loss, as follows [22]:

(5) $$L_{\mathrm{BCE}\text{-}\mathrm{Dice}}=L_{\mathrm{BCE}}+\left(1-\mathrm{Dice}\right)$$

Binary Cross-Entropy (BCE):

(6) $$L_{\mathrm{BCE}}=-\frac{1}{N}\sum_{i=1}^{N}\left[G_{i}\log\left(P_{i}\right)+\left(1-G_{i}\right)\log\left(1-P_{i}\right)\right]$$

Dice Loss:

(7) $$L_{\mathrm{Dice}}=1-\frac{2\sum_{i}P_{i}G_{i}+\epsilon }{\sum_{i}P_{i}+\sum_{i}G_{i}+\epsilon }$$

where:

- *N*: Total number of pixels.
- $P_{i}$: Predicted value of pixel *i*.
- $G_{i}$: Ground truth value of pixel *i*.
- ϵ: Stabilizing constant.

This combined loss enables both pixel-wise fidelity (BCE) and global region overlap (Dice), enhancing training stability and reducing segmentation inconsistencies.

### 4.4. Verification Through Partial Reconstruction

Finally, the masks segmented in the sinogram domain are subjected to filtered backprojection (FBP) to verify the lesion’s location in the original spatial domain. Although the reconstruction is not intended to recover the complete morphology, it allows confirmation that the region identified by the model corresponds to the true location, demonstrating the clinical utility of direct segmentation from sinograms.

#### Comparative Analysis

To evaluate the model’s performance, the three input configurations were compared using quantitative metrics and qualitative inspection of the obtained segmentations and their reconstructions.

The analysis focused on three main aspects: (i) the model’s ability to accurately localize lesions, (ii) the impact of the source image type (mammography or DBT) on the achieved performance, and (iii) the clinical feasibility of performing segmentation without reconstructing the original volume.

Finally, the results are interpreted in light of the inherent limitations of DBT, including potential artifacts and limited data availability, as well as the potential of segmented sinograms as an efficient alternative for lesion detection and localization in clinical settings.

## 5. Results and Comparison

The experimental results obtained by evaluating the proposed model under different input configurations are presented below, along with a comparison to a reference model based on YOLOv5x. This section is organized into three parts: (i) the description of the quantitative results using performance metrics; (ii) the qualitative analysis, including partial reconstruction from sinogram masks to validate the spatial localization of lesions; (iii) the comparison with the YOLOv5x model, highlighting the differences between the two approaches in the context of direct sinogram segmentation.

### 5.1. Quantitative Performance of the Model

The U-Net model was trained with three sinogram configurations: mammography, DBT, and combined data. Table 2 summarizes the Dice Scores obtained during the training and validation phases, along with the corresponding derived IoU values and approximate Precision and Recall. The Dice Score indicates the overlap between predicted and ground truth masks, while IoU was computed using the standard relationship $IoU=\frac{Dice}{2-Dice}$. Precision and Recall were approximated from the Dice Score.

The best results were achieved with mammography sinograms, yielding a Dice score of 0.90 on the validation set, indicating precise and consistent segmentations. In contrast, DBT sinograms showed more limited performance, with a Dice score of 0.70, although this was sufficient to delineate the anatomical region of interest. The combined configuration reached a Dice score of 0.84 during validation, suggesting some overfitting, possibly due to dataset heterogeneity.

### 5.2. Qualitative Analysis and Localization Assessment

**Figure 3:**
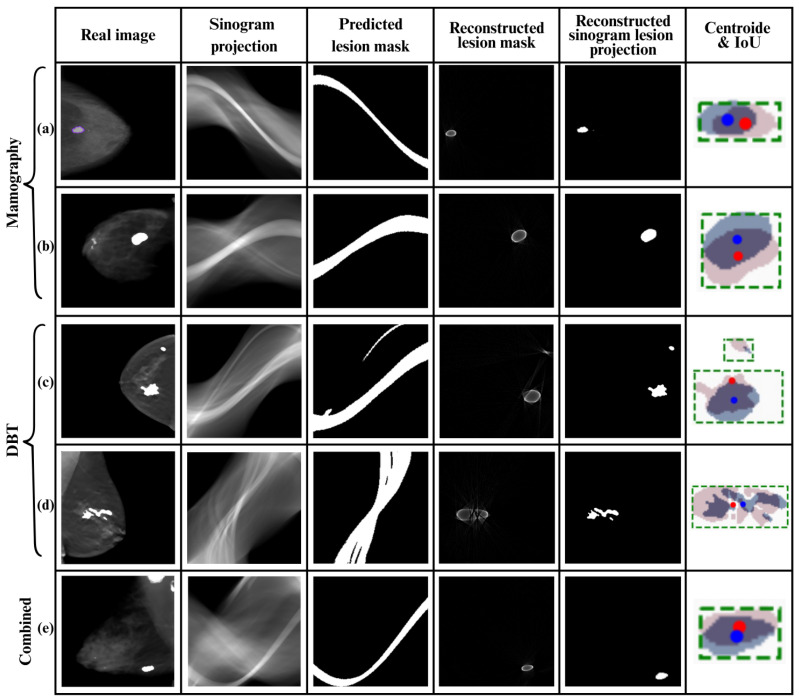
Visual comparison of segmentations and centroids across different input types. Panels (**a**) and (**b**) correspond to mammography, (**c**) and (**d**) to DBT, and (**e**) to the combined mammography and DBT input. The figure includes the visible lesion image, the sinogram, the predicted mask, the partial reconstruction, and the centroid overlay. Green dotted boxes indicate the region of interest around the lesion. Red dots represent the centroid of the ground truth mask, while blue dots represent the centroid of the predicted mask. The author’s own work.

Figure 3 shows examples of segmentation for each configuration. The masks obtained from mammography sinograms have well-defined edges, morphologically align with true lesions, and exhibit a low false-positive rate. For DBT sinograms, segmentation is less precise, with more diffuse edges, but it preserves the anatomical location of interest. This indicates that, although structural quality is lower in this domain, the angular information contained in DBT sinograms is sufficient to identify the lesion. The combined configuration produces robust segmentations and improves sensitivity to low-contrast lesions. However, the heterogeneity of the combined dataset results in greater variability in mask shapes.

#### 5.2.1. Reconstruction from Sinogram Masks

To assess the feasibility of localizing lesions without full reconstruction, FBP was applied to masks segmented by a model trained on DBT sinograms. The results shown in Figure 3 indicate that, despite the low resolution, the approximate shape and, more importantly, the location of the lesion can be accurately recovered. This capability is valuable for rapid clinical systems or those with computational limitations.

#### 5.2.2. Localization Assessment Using Centroids

One of the main objectives of this study is to provide a tool that not only segments breast lesions but also accurately identifies the anatomical region of interest. This type of localization is essential for identifying suspicious areas during clinical readings and for assisting automated pre-screening systems or reconstruction guidance.

However, segmentations directly generated from sinograms may exhibit irregular or blurred contours, which can negatively affect overlap-based metrics such as IoU. For this reason, centroid analysis was incorporated to robustly evaluate spatial accuracy, independently of contour morphology.

Likewise, sinograms efficiently preserve the angular information of the original object because they are constructed from the Radon Transform. This property ensures the calculated centroid and maintains high spatial fidelity to the true lesion location, despite deformations in the segmented mask.

To validate this hypothesis, the following steps were implemented:

1. Centroid calculation: The Euclidean distance between the centroids of the ground truth and predicted masks was computed.
  To quantify the spatial correspondence between the true and segmented lesions, the centroid of each binary mask *M* was calculated as:
  (8) $$\bar{y}=\frac{1}{N}\sum_{(y,x)\in M}y,\;\bar{x}=\frac{1}{N}\sum_{(y,x)\in M}x$$
  where *N* is the number of pixels belonging to the lesion (where M(y,x)=1).
2. Distance between ground truth and predicted centroids: The Euclidean distance between the centroid of the ground truth mask $\left({\bar{y}}_{r},{\bar{x}}_{r}\right)$ and the segmented mask $\left({\bar{y}}_{s},{\bar{x}}_{s}\right)$ was calculated as:
  (9) $$d=\sqrt{{\left({\bar{y}}_{r}-{\bar{y}}_{s}\right)}^{2}+{\left({\bar{x}}_{r}-{\bar{x}}_{s}\right)}^{2}}$$
3. Spatial overlap percentage: To express the overlap as a percentage, the distance was normalized by the image diagonal $D=\sqrt{H^{2}+W^{2}}$, yielding:
  (10) $$\mathrm{Overlap}(\%)=\max\left(0,100\cdot (1-d/D)\right)$$

Values close to 100% indicate strong alignment between the masks, while values near 0% indicate significant spatial discrepancy. The analysis was complemented with a cropped IoU, focused exclusively on the lesion region, allowing a fairer evaluation in images with multiple masses or a large background area. These quantitative results are summarized in Table 3, providing centroid distances, location match percentages, and IoU within the lesion region for each image and input type. These metrics were also supported by visual analysis (see Figure 3), which shows the overlap between the lesions in the original image (in red) and the automatically segmented masks (in blue), as well as the comparison of their respective centroids. Green dotted boxes highlight the region of interest around each lesion. The visual consistency between both locations supports the validity of this approach. Also, filtered backprojection (FBP) was applied to the segmented masks, and the resulting contours were reintegrated into the original images. This verification reveals that the regions identified by the masks, even in the sinogram domain, spatially correspond to the true lesions. These findings support the use of sinograms for localization tasks without requiring full volumetric reconstruction. For cases with multiple lesions, each lesion was analyzed separately to ensure the accuracy and validity of the spatial overlap metrics.

The analysis by input configuration reveals distinct behaviors depending on the sinogram’s origin. For mammography (a and b), segmentations showed morphology consistent with the true lesion, with IoU values above 0.42 and spatial overlap exceeding 97.9%, indicating proper anatomical alignment. For DBT-derived sinograms (c and d), although the IoU was considerably lower, especially for small lesions, the model maintained highly accurate localization (over 97.8%), demonstrating that the angular information contained in these sinograms is sufficient to preserve lesion location even when the segmented shape is irregular. Finally, the combined configuration (e) achieved the best overall performance, reaching the smallest centroid distance (2.83 px) and the highest location overlap (99.11%). These results suggest that combining mammography and DBT sinograms provides complementary information.

These findings reinforce the value of using sinograms as direct inputs for segmentation, prioritizing localization over morphological fidelity, particularly in clinical contexts where efficiency is desired, such as assisted diagnosis or automated detection.

The quantitative and qualitative results indicate that, even when morphological fidelity is low, the model successfully maintains precise lesion localization. This is particularly important in scenarios where identifying suspicious regions is prioritized over exact shape recovery. For mammography sinograms, the model achieved a balanced performance between segmented shape (IoU) and spatial localization based on the centroid. On the other hand, DBT sinograms exhibited lower IoU values, while localization accuracy remained high, demonstrating that the angular information in the sinogram is enough to preserve the lesion’s anatomical position. The combined configuration proved to be the most effective, achieving the highest accuracy. This clearly shows that using both mammography and DBT sinograms enhances the model’s robustness and makes it reliable for localization tasks.

### 5.3. Comparative Analysis of YOLOv5x and U-Net Models

While the previous results highlight the U-Net’s superiority in spatial accuracy and anatomical localization, especially in scenarios with multiple lesions or irregular shapes, it is also essential to evaluate its practical feasibility in real clinical settings. In these environments, factors such as inference time, computational cost, and scalability are critical for the effective deployment of an AI model.

For this reason, a direct comparison was made with YOLOv5x, a widely adopted reference model for detection tasks due to its efficiency and speed. This model uses bounding boxes rather than pixel-wise segmentations, offering a lighter but less detailed approach.

Regarding the comparison with YOLOv5x, it is important to clarify that YOLOv5x was trained using the same original DBT images (272 images). However, for YOLOv5x, an additional 1000 images were generated through data augmentation to expand the dataset. In contrast, for U-Net, the model was trained with the 272 annotated images, and data augmentation was applied internally during training. Both methods used the same dataset split for training, validation, and testing, ensuring a fair comparison. This setup highlights the differences between segmenting in the original image domain (YOLOv5x) versus directly in the sinogram domain (U-Net), showing the advantages of working with aggregated projection information while maintaining a comparable number of cases for evaluation.

In a previous study [23], YOLOv5x was trained on the same DBT dataset and evaluated on its ability to detect breast lesions in reconstructed images. Although YOLOv5x is traditionally expected to have shorter inference times due to its architecture, in this study the U-Net model demonstrated significantly lower inference times. This difference can be attributed to specific characteristics of the experimental setup, image size and type, and the optimizations applied to each model.

A quantitative comparison between the two models is presented below.

As shown in Table 4, U-Net requires less training time, has a lighter architecture, and achieves competitive segmentation results with reasonable inference times. These characteristics make it a viable option for real-time clinical applications or devices with limited resources, where both speed and accuracy in anatomical lesion localization are critical.

One of the most relevant methodological differences between the approaches evaluated in this study lies in how breast lesion localization is represented. While YOLOv5x uses bounding boxes (bounding boxes), the U-Net architecture generates pixel-level segmentation masks, allowing for a more precise and anatomically coherent morphological representation.

This distinction has important clinical implications:

- Bounding boxes: Due to their rectangular nature, they encompass both the lesion and surrounding healthy tissue. This characteristic can introduce diagnostic ambiguity, particularly in cases with small lesions, irregular contours, or blurred edges.
- U-Net masks: Conform more accurately to the true shape of the lesion, enabling:
  - More precise planning of invasive procedures, such as biopsies.
  - More reliable longitudinal follow-up across studies.
  - Accurate quantification of the affected area, essential for assessing disease progression or therapeutic response.

The qualitative results further reveal that U-Net segmentations:

- Capture the edges and true morphology of the lesion with higher accuracy.
- Reduce the inclusion of irrelevant regions, thereby decreasing the risk of false detections and providing a more precise and useful visual guide for clinicians, facilitating decision-making.

Although U-Net training requires more detailed manual annotations, this results in segmentations with higher anatomical accuracy. In contrast, YOLOv5x uses simpler annotations, which reduces the morphological fidelity of its outputs.

**Figure 4:**
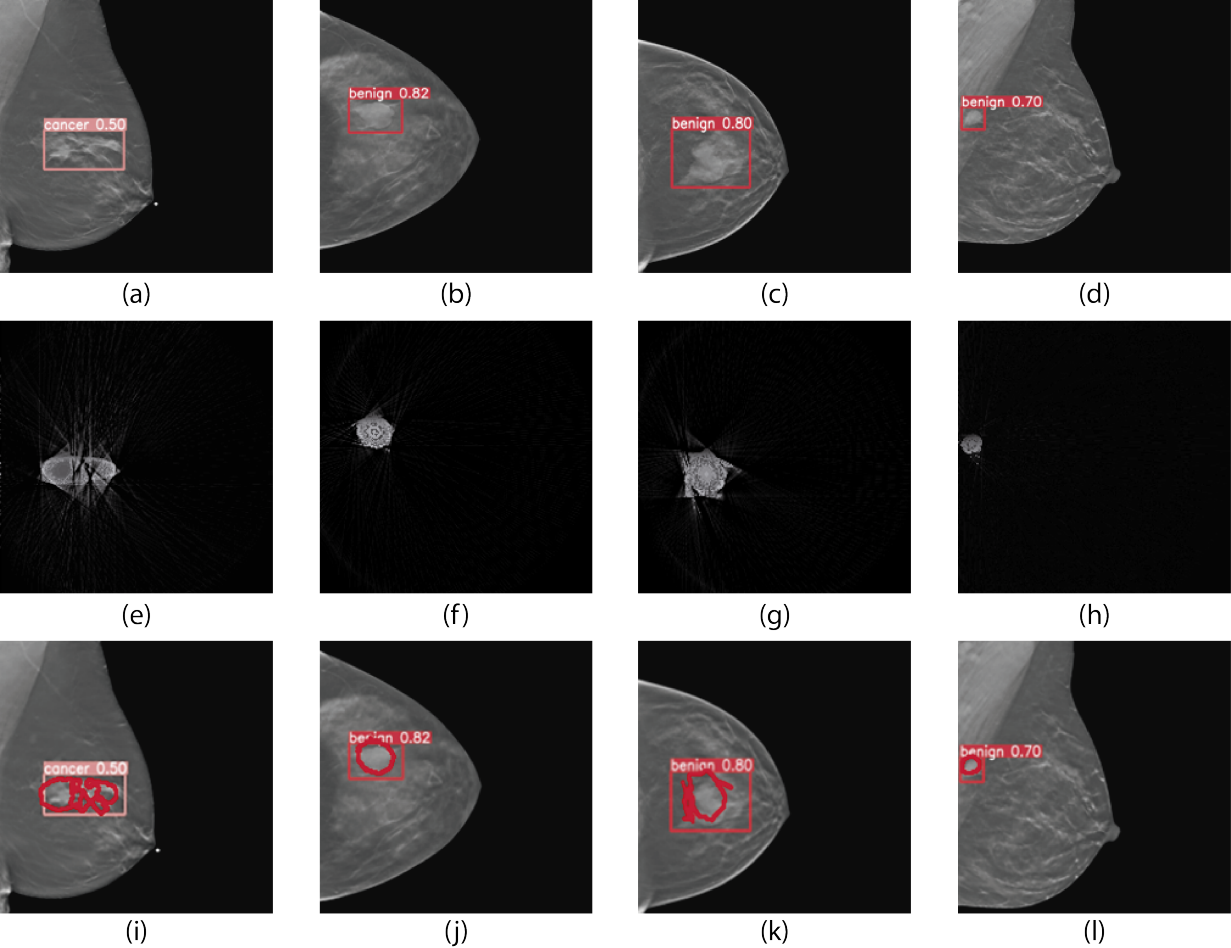
Qualitative overlap between masks segmented by U-Net and bounding boxes generated by YOLOv5x (the author’s own work). The images (**a**–**d**) show the DBT image with the prediction generated by YOLOv5x; (**e**–**h**) show the reconstruction obtained from the sinogram-segmented mask; (**i**–**l**) show the overlay of the bounding box produced by YOLOv5x with the mask generated by U-Net. Red bounding boxes represent the predictions by YOLOv5x, while red masks correspond to the segmentation produced by U-Net. This visualization allows direct comparison of U-Net segmentation with YOLOv5x localization, highlighting spatial correspondence and discrepancies.

In general, U-Net is better suited to clinical applications that require high precision, such as therapeutic planning or individualized follow-up. Conversely, YOLOv5x is ideal for preliminary or triage tasks, where speed and real-time implementation are prioritized. Figure 4 presents a visual comparison between the two approaches.

## 6. Discussion and Results

Unlike previous studies focused on reconstructed mammograms or DBT volumes, this work emphasizes direct segmentation of sinograms, a relatively unexplored domain despite its computational advantages and preservation of the original acquisition information. While established methods, such as 3D-Mask R-CNN applied to DBT or advanced U-Net variants in mammography and ultrasound, operate on images where lesion morphology is explicit, sinograms represent integral projections that lack clear edges and intuitive visual patterns. This contrast introduces a significantly greater challenge and requires models capable of learning indirect representations of the anatomical structure.

In this context, the main contribution of this study is not to surpass the morphological accuracy of approaches based on reconstructed images, which would be methodologically unfair given the inherently different nature of the sinogram domain, but rather to demonstrate that lesions can be localized with high precision without reconstruction. This approach offers key benefits: reduced processing time, lower sensitivity to reconstruction artifacts, and preservation of primary signals that may be useful for computer-aided diagnostic systems.

We explicitly acknowledge that our approach relies on sinograms generated via the Radon transform from reconstructed mammography or DBT slices, rather than using direct projection data from the scanner. This distinction may affect the fidelity of the projections compared to raw acquisitions. Additionally, external validation and generalization to different scanners or acquisition protocols have not yet been performed, which may impact the applicability of our method in diverse clinical settings. Other factors, such as lesion size, tissue density, breast composition, and the annotation burden required for training, may also influence model performance. Recognizing these limitations highlights directions for future research, including validation on multi-institutional datasets, evaluation of robustness to patient and scanner variability, and strategies to reduce manual annotation requirements.

The results show that, even when the segmented morphology in sinograms is irregular, as expected due to the non-visual nature of these representations, the spatial localization derived from centroids is consistent and stable, achieving accuracies that support its clinical utility. Additionally, combining mammography and DBT sinograms exhibits more robust behavior than using each modality individually, suggesting that multi-source sinograms contain complementary information that enhances localization.

Compared to studies based on reconstructed images reporting higher metrics, the differences observed in this study are justified by the more challenging conditions of the analyzed domain and, above all, by the shift in objective: while traditional models optimize anatomical segmentation, here the focus is on direct spatial localization in the sinogram. This methodological shift constitutes a novel contribution, moving the focus from reconstruction to exploitation of the acquisition domain.

Additionally, the quantitative performance confirms the approach’s feasibility: mammography-derived sinograms achieved high performance (94% in training, 90% in validation), while DBT sinograms showed lower performance (77% and 70%), likely due to tissue overlap, structural noise, and limited availability. Although the combined configuration improved upon using DBT alone, it did not reach mammography levels, suggesting that multimodal integration requires more advanced fusion techniques or adaptive weighting.

Regarding clinical utility, the results indicate that direct segmentation of sinograms is both feasible and efficient. The reduced computational load, absence of reconstruction artifacts, and centroid stability (reinforced through partial FBP reconstructions) support the potential of this approach in scenarios where speed is critical, such as triage systems or automated pre-screening.

Finally, this work also highlights relevant limitations, now explicitly acknowledging that the sinograms were generated from reconstructed slices rather than direct scanner projections. The most significant is the scarcity of high-quality DBT sinograms, which limits model training and generalization. In addition, segmentation requires precise manual annotations, increasing data preparation costs. External validation across different scanners and protocols, sensitivity to lesion size and tissue composition, and the annotation burden represent important directions for future investigation.

Future directions: Building on the present findings, several avenues can be pursued to further enhance the methodology and clinical applicability:

- Advanced multimodal fusion: Integrating information from mammography, DBT, and potentially other imaging modalities through adaptive weighting, attention mechanisms, or transformer-based fusion could improve localization and robustness.
- Automated or semi-automated annotation strategies: Implementing annotation-efficient approaches, such as weak supervision, self-supervised learning, or synthetic data generation, could reduce manual burden and accelerate dataset expansion.
- Cross-scanner and protocol generalization: Evaluating the approach on diverse scanners, acquisition settings, and patient populations will strengthen its clinical reliability and external validity.
- Sensitivity analysis: Studying the influence of lesion size, density, and breast composition on model performance may guide adaptive pre-processing or model customization for specific patient subgroups.
- Integration with clinical workflows: Future work could explore embedding sinogram-based segmentation into real-time pre-screening systems, triage tools, or computer-aided diagnosis pipelines, leveraging the computational efficiency and direct projection analysis offered by this approach.

Overall, the findings expand the understanding of the potential of sinograms not only for reconstruction tasks but also for direct segmentation and localization. This establishes a methodological framework that can serve as a foundation for future research in hybrid segmentation, multimodal fusion, early detection in the projection domain, and clinical deployment strategies.

## 7. Conclusions

This study demonstrated the feasibility of segmenting breast lesions directly from sinograms, bypassing the volumetric reconstruction step. Unlike most previous works focused on reconstructed DBT images or contrast enhancement, this research addresses, for the first time, direct segmentation in the sinogram domain. The results showed that the model trained on mammography-derived sinograms achieved the best performance in both segmentation and localization, outperforming DBT-based sinogram configurations and the combined dataset.

Although DBT sinograms offered lower morphological fidelity, they preserved angular information, enabling precise anatomical localization, as validated by partial reconstruction and centroid analysis. Compared to traditional detection methods, such as YOLOv5x applied to reconstructed images, the U-Net implemented on sinograms provided more accurate localization of suspicious regions, highlighting its potential for CAD systems aimed at early detection and for clinical environments with limited resources.

Overall, the findings of this work expand the possibilities of sinogram analysis in mammography and tomosynthesis, showing that direct segmentation is not only technically feasible but also clinically relevant. Future work includes exploring advanced multimodal fusion strategies and increasing the availability of DBT sinograms to improve model generalization and robustness. These advances could support the development of hybrid tools for efficient early detection and localization directly in the projection domain.

## Figures and Tables

**Table 1:** Related work on networks applied to breast images and sinograms.

Author/Year [Ref]	Network Type	Modality	Problem Addressed	Method/Contribution	Metrics
Zhou et al., 2021 [8]	3D-Mask R-CNN	DBT	Direct 3D mass segmentation	3D network capturing volumetric structure	Sensitivity 90%, 0.8 FP/image
Fan et al., 2022 [9]	AAU-Net (U-Net with spatial + channel attention)	Ultrasound	Segmentation of complex tumors	Dual attention mechanism enhancing delineation	Improved Dice and Accuracy
Punn et al., 2021 [10]	RCA-IUNet (U-Net + Inception + cross-attention)	Ultrasound	Tumors of multiple sizes	Multiscale integration with Inception-like modules	High clinical accuracy
Haq et al., 2021 [11]	U-Net 3+	Mammography (INbreast)	2D lesion segmentation	Dense connections + multiscale aggregation	Dice 98.47%
Minh et al., 2024 [11]	UGGNet (U-Net + VGG16)	Ultrasound	Binary lesion classification	Fusion of U-Net with VGG16 for robust features	Accuracy 78.2%
Abuowaida et al., 2025 [12]	UltraSegNet (U-Net with multiscale attention)	Ultrasound (BUSI)	Segmentation of varied tumors	Spatial attention with multiscale fusion	Dice 91.1%, Accuracy 91.5%
Ruiz Muñoz et al., 2024 [13]	ResNet18/ResNet50	DBT Sinograms	Reconstruction-free classification	Direct sinogram analysis for lesion differentiation	Accuracy/Sensitivity/F1: 94.96%
Aootaphao et al., 2023 [14]	U-Net	Micro-CT sinograms	Artifact reduction	Direct pre-reconstruction segmentation	Enhanced image quality and reduced artifacts

**Table 2:** Performance metrics obtained during training and validation for each input configuration.

Data Type	Dice Score (Train)	Dice Score (Validation)	IoU (Train)	IoU (Validation)
Mammography	0.94	0.90	0.97	0.82
Tomosynthesis	0.77	0.70	0.87	0.54
Combined	0.94	0.84	0.97	0.71

**Table 3:** Localization assessment.

Image	Centroid Distance (px)	Location Match (%)	IoU in Lesion Region (%)
Mammography (a)	5.86	98.15	0.42
Mammography (b)	6.65	97.90	0.44
DBT (c) Lesion 1	98.20	0.04	0.04
DBT (c) Lesion 2	98.30	0.54	0.54
DBT (d)	6.74	97.87	0.32
Combined (e)	2.83	99.11	0.53

**Table 4:** Comparison between YOLOv5x and U-Net architectures.

Characteristic	YOLOv5x	U-Net
Architecture	Deep network with 322 layers	Segmentation architecture with 41 layers
Number of parameters	∼86 million	∼31 million
Input dimension	224 × 224 pixels	224 × 224 pixels
Preprocessing time	1.8 ms	2–5 ms
Inference time per image	920.5 ms	20–50 ms
Post-processing time	0.1 ms	2–5 ms
Total training duration	59.1 h	4 h
Performance metrics (validation)	Not reported	Loss = 0.2750; Dice = 0.8423

## Data Availability

The data used in this study are openly available: mammography images from CBIS-DDSM (https://www.cancerimagingarchive.net/collection/cbis-ddsm/) and DBT images from Breast Cancer Screening DBT (https://www.cancerimagingarchive.net/collection/breast-cancer-screening-dbt/), both last accessed on 8 December 2025.
